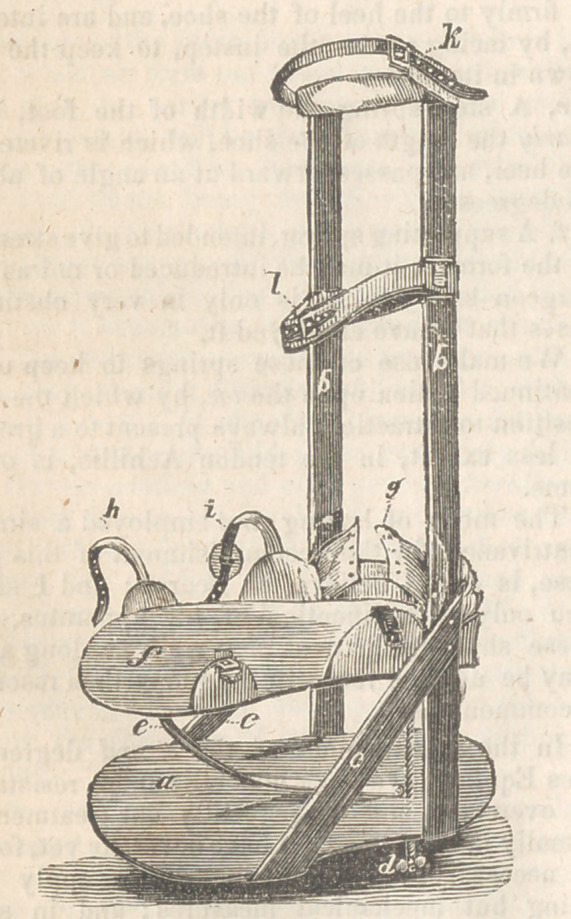# A Lecture on Loxarthrus, or Club Foot

**Published:** 1839-03-16

**Authors:** 


### BIBLIOGRAPHICAL NOTICE.

*A Lecture on Loxarthrus, or Club Foot.* By THOMAS D. MÜTTER, M. D., Lecturer on Surgery, &c. Philadelphia: 1839. 8vo. pp. 104.

DR. MÜTTER has, since the 1st of June, 1838, operated, by the division of the tendo-Achillis, upon twenty-eight cases of club foot. His success with these cases has induced him to publish a lecture on the subject,—chiefly, we presume, with a view to present certain modifications of his own in the apparatus for the treatment of the deformity. In No. 12 of Vol. I., we published a history and description of the operation for club foot by the division of the tendo-Achillis, by Dr. Detmold, of New York. Referring our readers to this paper, we shall pass to such points in Dr. Mütter's lecture as require special consideration. His remarks on the *prognosis* of the affection are worth noting.

"The *age* of the individual must also be held in view, when we form our prognosis. It is usually stated that the earlier we commence our treatment the greater will be the prospect of ultimate success; the muscles, ligaments, and even the bones themselves, being just after birth, *flexible* and *soft* and consequently more readily moulded into a proper shape. At first sight this advice appears reasonable enough, but you will find that whenever it becomes necessary from the character of the deformity to employ more force, or divide a tendon, great difficulty will be met with, if you follow it. In the first place, the *integuments* of a new-born child are so extremely delicate, that *excoriations* and often severe *ulcerations* are almost sure to be developed, even when the utmost care has been taken to prevent their appearance, by padding the instruments, and applying them as accurately as possible. We are of necessity, then, obliged to suspend our efforts for the relief of the defect, and wait until the foot is once more sound, and apparently able to support the pressure of our bandages without suffering.

In the second, the *legs* of a very young child are so *short* and *clumsy* that it is almost impossible to maintain a proper apparatus. It is constantly slipping, and of course can exert little or no influence upon the deformity, for the removal of which it is applied. The constant *flexion* of the limbs, and the frequent necessity for changing the child, are likewise obstacles to a proper action of our apparatus. In the *third*, the *nervous* system of very young infants is so susceptible, that the slightest causes are often sufficient to throw them into convulsions, or bring on fever. In a child of Mr. A., upon whom, in its third week, I applied my apparatus, after having previously divided the tendon Achillis in both feet, I was obliged to stop the treatment after the lapse of a day or two, in consequence of its fretting itself into a fever. There was probably not much suffering in this case, because I took great pains to make the bandages as soft as possible, and there was no excoriation; but the mere confinement of the feet was sufficient to cause great disturbance of the whole system. For the reasons just stated, then, I always, when it is in my power to do so, postpone the commencement of the treatment until the child is five or six months old. We should never, provided the case be under our control, permit it to remain unremedied longer than the second year, for after this period the difficulties of the treatment are astonishingly increased.

From my own experience, I should say *decidedly*, that the *most favourable period* ranges between the sixth and eighteenth months. The integuments at this age are sufficiently firm to bear the requisite degree of pressure without suffering, while the *muscles*, *ligaments* and *bones*, are all susceptible of being easily brought to their normal position. I have also found, that when the child has been allowed to pass this period without treatment, the next best period ranges between two and eight; after which the case becomes more and more difficult, as year is added to year."

We shall extract the chapter on the treatment of Pes Equinus, which contains a description of a modified apparatus, of Dr. Mütter's invention.

We have availed ourselves to such an extent of Dr. Mütter's text, that we have little room left for critical remarks. The caption of the lecture is incorrect—the term *loxarthrus* not being synonymous with club foot, but a generic title for obliquities of joints, (from λοξος, oblique, and αρθρον, a joint.)

"We have lastly to speak of the proper treatment in Pes Equinus.

In this form of club foot the indication is generally extremely simple; the defect residing almost exclusively in the tendon Achillis, its elongation is nearly all that is required to effect a cure, except in cases of long standing.

The first degree, where the heel is but slightly elevated, when met with at birth, may always be cured in a short time, by the use of a properly contrived stretching apparatus, which should be worn day and night, until the object in view is attained. The instrument which I employ, is exceedingly simple. It consists of a footboard furnished with four mortices, through which the tapes of a gaiter are passed; of a strap attached to its point; of leg-irons which extend to the knee, and of a buckle attached to the anterior portion of the strap of the leg-irons.

*A*, Foot-board, which is attached to the leg irons by a wire joint, so that its angle of inclination may be varied at pleasure.

*B*, Leg-irons passing up to the knee.

*C*, Strap to attach the leg-irons to the limb.

*D*, Buckle intended for the toe-strap.

*E*, Toe-strap, by which the toe is elevated.

The gaiter having been adjusted to the ankle, we place the sole of the foot *flat* upon the board, and fasten it down by tying the tapes securely. We next fasten the strap of the leg-irons, and then pass the toe-strap through the buckle. Every day or two we take up a hole, and as we elevate the *toe*, it is evident the *heel* must descend. This simple contrivance answers perfectly, when the force to be applied is not very great. Having brought the heel down, we employ a straight shoe furnished with instep straps, which should be worn day and night by the child, until all tendency to retraction of the tendon Achillis has disappeared.

In children between the ages of one and six, the same apparatus will answer in time; but I would not hesitate in such cases about the division of the tendon in fault. It should be severed at once, and in the course of ten days the child may begin to walk, in shoes furnished with instep-straps, to keep the *heel down* in contact with the sole. When a person is advanced in life, and labours under this degree of Pes Equinus, he can only be relieved, *in a short time*, by a section of the tendon Achillis. Cases are reported in which the spring shoe of Scarpa, the sabot of Venel, and other contrivances, have, with much suffering, and after a long time, brought down the heel; but do not, I beg of you, subject your patients to any such treatment; divide the tendon in all such cases. After its division, you may employ the simple stretcher just described, and when the parts are sufficiently united, and the heel down, give your patient a shoe such as I now show you.

*A*, A common high quartered shoe, fitting closely around the ancle, and lacing from the toe up.

*ab*, Left instep strap.

*cd*, Right instep strap. These straps are stitched firmly to the heel of the shoe, and are intended, by lacing across the instep, to keep the *heel* down in its place.

*e*, A steel spring the width of the foot, and *nearly* the length of the shoe, which is riveted to the heel, and passes forward at an angle of about 35 degrees.

*f*, A supporting spring, intended to give strength to the former; it may be introduced or not as the surgeon sees fit. It is only in very obstinate cases that I have employed it.

We make use of these springs to keep up a continued action upon the *toe*, by which the disposition to retraction, always present to a greater or less extent, in the tendon Achillis, is overcome.

The merit of having *first* employed a similar contrivance, for the accomplishment of this purpose, is due, I believe, to Scarpa; and I show you only a modification of his apparatus. In these shoes the patient may walk as long as it may be deemed requisite; he may then resort to a common one.

In the management of the *second* degree of Pes Equinus, we have generally more resistance to overcome, and consequently our treatment is usually more tedious. I have never, as yet, found it necessary, when called early, to apply any thing but mechanical measures; and in such cases, the treatment recommended as proper in the early stages of the *first* degree, will generally effect a cure.

In persons between the first and sixth year, I

would advise you not to waste time in attempts to *stretch* the tendons; for although this, sooner or later, will succeed in bringing the foot to a proper position, yet you will save your patient much suffering, and yourself much trouble, by their division. The subsequent treatment is precisely similar to that recommended in *old cases* of the *first degree.*

When called to a case, some what advanced, you must calculate upon a tedious treatment. To effect a cure it is essential to divide the tendon Achillis, and often the fascia plantaris, which is here remarkably dense and rigid. It is in these cases too that we are so often obliged to divide the tendons of the *toes*, some of which are almost invariably so much distorted, that it would be impossible to employ a proper shoe, until they are brought to a more normal position. In these cases, I have also found, that the simple stretcher already described, did not possess sufficient force for the elevation of the toes; and besides this, it is necessary, from the great deformity of the foot, to employ a more complicated apparatus than a gaiter, to fix it upon the foot-board. It is, moreover, of great importance, in cases where it becomes necessary to employ much force, to have an apparatus so constituted that it may be *regularly* and *gradually* kept up, and increased or diminished at will, without deranging the whole dressing. The apparatus which I show you, and which is of my own invention, fully answers all these purposes.

*a*, An iron plate, the eighth of an inch in thickness, and a little longer than the foot.

*bb*, Leg-irons extending to the knee.

*cc*, Strips of iron about an inch wide, which serve to strengthen the attachment between the leg-irons and the lower plate.

*d*, A screw, which, passing through the lower plate, is firmly attached to the heel of the upper, and is so threaded, that when we force the nut *on*, the heel is necessarily depressed. The advantage of this screw consists in the *regularity* and *steadiness* of its action.

*e*, A strong steel spring, which passes upwards from the heel of the lower plate, and is intended to act upon the *toe* of the upper, while the *heel* is *depressed* by the screw. The strength of this spring must, of course, depend upon the nature of the case.

*f*, An iron plate, of the dimensions of the lower. It should be a little wider than the foot, and furnished with a *heel-piece* about two inches high, which prevents the heel from slipping backwards; and with side pieces, two on each side, which prevent *lateral* slipping of the foot, and also serve for the attachment of the straps and buckles, which are intended to fasten it securely.

*g*, Instep-straps, which lace in front, and are firmly stitched to the heel-piece of the upper plate. These should never be forgotten, as they *fix the heel.*

*h*, A strap which passes across the toes, and laces on the inside of the foot.

*i*, A strap which passes across the instep, fastens on the inside of the foot, and assists in keeping the sole in contact with the plate.

*k*, Strap just below the knee, intended to attach the leg-irons.

*l*, One just below the calf, for the same purpose.

The tendons having been divided, the wound closed, and the gaiter adjusted to the ankle, we next place the foot upon the upper plate, which, in order that it may accommodate itself to the inclination of the sole, must be *depressed* in *front*, while the *heel* is allowed to *ascend.* We then tie the gaiter tapes beneath the upper plate, by passing them through the mortices, or even by carrying them over the *sides* of the plate. Next, lace the instep-straps, and buckle those which cross the anterior portions of the foot; finally, fasten the leg-irons. The foot is thus firmly secured, and we commence at once the depression of the heel, by turning the screw until the patient complains of uneasiness; we then fix it at this point. The next day another turn of the screw may be given until by degrees we bring the plates to the relative position shown in the cut. The angle formed between the foot and the leg, will have become by this time nearly a *right* one, and the patient will have pretty good use of the member in a very short time.

This apparatus should only be removed for the purpose of bathing the foot; and wherever there is much pressure, cotton wadding, or an air cushion, must be placed between the skin and the instrument. After it has accomplished the end for which it was employed, the shoe with a spring on the sole may be substituted for it, and worn as long as it may be deemed necessary.

In Pes Equinus of the third degree, (Talapes

Equinus Verus of Mr. Little) occurring at birth, I advise you to divide the tendon Achillis at once, and employ afterwards the simple stretcher already described.

In the same defect, met with in children between the first and sixth year, the tendon should also be divided, and in the course of two or three weeks, the child made to walk in the spring shoes already described, until the retraction of the heel is entirely overcome. For the depression of the heel, the apparatus just recommended for infants may be employed.

When the case has been neglected until the individual is advanced in life, the treatment recommended in similar cases of the *second* degree of Pes Equinus must be employed.

In all these cases, the usual general treatment, such as frictions, bathing, &c., is to be pursued."

## Figures and Tables

**Figure f1:**
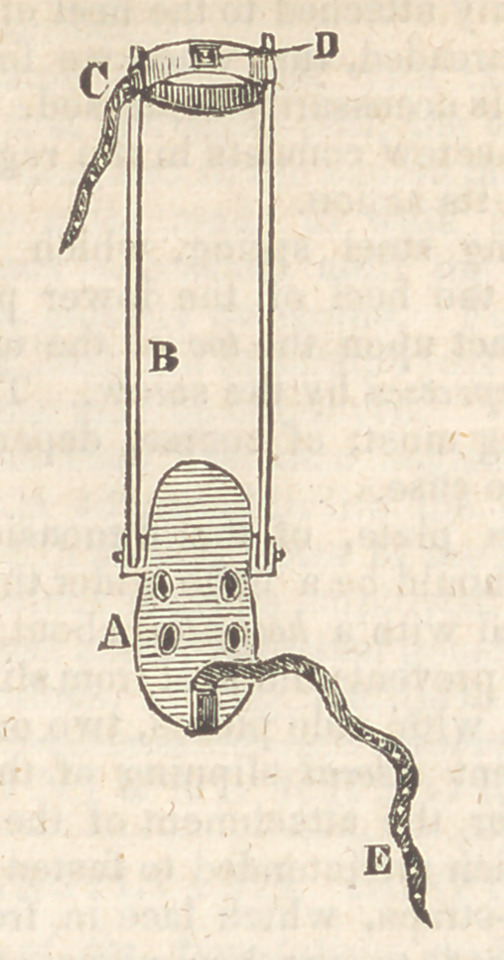


**Figure f2:**
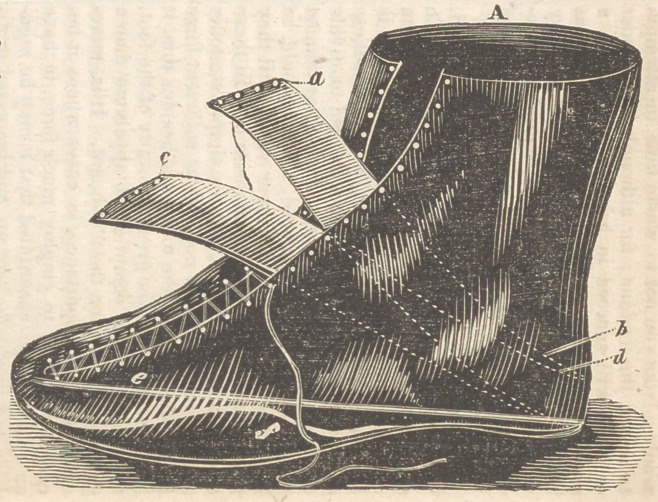


**Figure f3:**